# Impact of clear aligner therapy on masticatory musculature and stomatognathic system: a systematic review conducted according to PRISMA guidelines and the Cochrane handbook for systematic reviews of interventions

**DOI:** 10.1186/s12903-024-04029-8

**Published:** 2024-03-19

**Authors:** Sultan Abdulrahman Almalki, AlBandary Hassan Al Jameel, Inderjit Murugendrappa Gowdar, Akshayraj Langaliya, Sunil Kumar Vaddamanu, Marco Di Blasio, Gabriele Cervino, Giuseppe Minervini

**Affiliations:** 1 Department of Preventive Dental Sciences, College of Dentistry, Prince Sattam Bin AbdulAziz University, Al-kharj, 11942 Saudi Arabia; 2 Department of Periodontics and Community Dentistry, College of Dentistry, King Saud University, P.O. Box 60169, Riyadh, 11545 Saudi Arabia; 3 Department of Preventive Dental Sciences, College of Dentistry, Prince Sattam Bin AbdulAziz University, Al-kharj, KSA 11942 Saudi Arabia; 4 Department of Conservative Dentistry and Endodontics, AMC Dental College and Hospital, Ahmedabad, India; 5 Department of Dental Technology College of Applied Medical Siecence, King Khalid University, Abha, Saudi Arabia; 6 Department of Medicine and Surgery, University Center of Dentistry, University of Parma, Parma, 43126 Italy; 7 School of Dentistry, Department of Biomedical and Dental Sciences and Morphofunctional Imaging, University of Messina, via Consolare Valeria, 1, Messina, 98125 Italy; 8 Saveetha Dental College and Hospitals, Saveetha Institute of Medical and Technical Sciences (SIMATS), Saveetha University, Chennai, Tamil Nadu, India; 9 Multidisciplinary Department of Medical-Surgical and Odontostomatological Specialties, University of Campania “Luigi Vanvitelli”, Naples, 80121 Italy

**Keywords:** Temporomandibular disorders, Masticatory musculature, Orthodontic treatment, Aligners, Bite force, Orthodontics, Oral health

## Abstract

**Background:**

Clear aligner therapy has gained popularity as a minimally invasive orthodontic treatment option. However, its impact on the masticatory musculature and the stomatognathic system is an area of growing interest, as it involves the adjustment of occlusion and tooth movement. This systematic review aims to comprehensively assess and synthesise existing evidence regarding the influence of clear aligner therapy on the masticatory musculature and the stomatognathic system.

**Methods:**

An exhaustive search was performed on electronic databases that adhered to PRISMA guidelines. Clinical studies that evaluated the impact of patients receiving aligner orthodontic treatment on the muscles of the mastication and stomatognathic systems were included. A standardised data extraction form was devised for relevant variables. Two reviewers extracted the data variables. ROB-2 was used for bias evaluation in the selected studies.

**Results:**

A total of six studies met the inclusion criteria. The wearing of clear aligners significantly impacted the muscles of mastication. Muscle activity and discomfort showed a significant alteration in the initial days of appliance placement. but this observation was temporary, with no significant changes thereafter in subsequent follow-up. Bite force reduction was also noted. All the studies evaluated showed good methodological quality.

**Conclusion:**

The review found that aligned orthodontic treatment may have a variable impact on muscles of mastication, with a potential for initial exacerbation of symptoms followed by possible improvement. However, due to the limited number of studies and their heterogeneous nature, further robust research is recommended to fully understand the relationship between aligned orthodontic treatment and masticatory muscles.

## Introduction

Clear orthodontic aligner treatment has become an integral part of dental healthcare due to the increasing demand for improved dental aesthetics and function [1]. This form of treatment employs devices such as aligners or braces to adjust the position of teeth, thereby enhancing the alignment and overall oral health of individuals. However, alongside the pronounced benefits, there have been concerns regarding the potential adverse effects of orthodontic treatment [2] (Table 1). One area of particular interest has been the association between aligned orthodontic treatment and the development of TMDs. TMDs comprise a group of conditions characterised by pain and dysfunction in the TMJ and the muscles of mastication [3]. These disorders can significantly impact an individual’s quality of life, causing symptoms such as facial pain, headaches, and difficulties in chewing and speaking [4–6]. The aetiology of TMDs is multifactorial, with factors such as trauma, stress, systemic diseases, malocclusion, and potentially orthodontic treatment playing contributory roles.

**Table 1:** Abbreviations utilised in the review

Abbreviation	Full Form
TMD	Temporomandibular Disorder
OBC	Oral Behavior Checklist
TA	Anterior Temporal Muscle
MPP	Mandibular Postural Position
MVC	Maximal Voluntary Contraction
SCM	Sternocleidomastoid
MM	Masseter Muscle
sMMA	Sleep-time Masticatory Muscle Activity
SB	Sleep Bruxism
sEMG	Surface Electromyography
PCA	Passive Clear Aligners
MMA	Masticatory Muscle Activity
OD	Occlusal Discomfort
CAT	Clear Aligner Therapy
3D	Three-Dimensional
TMJ	Temporomandibular Joint

Research in the scientific domain indicates a potential influence of orthodontic treatment and occlusal devices on the activation of masticatory muscles [7–9]. In the context of patients diagnosed with myofascial pain syndrome, the application of multi-bracket devices for tooth realignment appears to yield a positive impact on masticatory muscle discomfort, albeit without completely eliminating the symptoms [10]. Additionally, the utilisation of removable retainer appliances that accommodate dental coverage has been found to decrease basal activity within the anterior temporalis muscle [7, 11–23].

In cases involving internal derangement of the temporomandibular joint, the introduction of an occlusal device has been shown to affect the activation patterns of the masseter and anterior temporalis muscles [24–29]. Moreover, a month-long treatment regimen with occlusal splints resulted in decreased masticatory muscle fatigue during maximal voluntary clenching in individuals with myofascial pain syndrome [30–32]. Given the multifactorial etiology of TMDs [11, 33], muscle responses are dependent not only on dental relationships but also on a multitude of other factors.

The heterogeneous outcomes observed when orthodontic treatment is employed as the sole therapeutic intervention for TMDs can likely be attributed to the complex, multifactorial nature of TMDs [8]. A myriad of elements, including parafunctional habits, occlusal interferences, and psychological components, have been implicated in the genesis and persistence of TMDs [9]. Orthodontic treatment, while addressing some aspects, might not comprehensively cater to these factors, thereby resulting in suboptimal management of TMDs. However, a combination of orthodontic treatment with other therapeutic modalities, such as physical therapy, pharmacotherapy, and behavioural therapy, might yield superior outcomes. This is likely due to the comprehensive nature of a multimodal approach that addresses all contributing factors to TMDs [34–42].

Moreover, the vast majority of the existing literature focuses on traditional orthodontic treatments, such as fixed braces, with limited research examining the potential effects of more recent advances in orthodontics, such as aligner-based treatments [30, 33–35]. Characteristics of similar studies on the impact of clear aligner therapy on the masticatory musculature and stomatognathic system were assessed to contextualize the contributions of this systematic review. The selected studies, in alignment with PRISMA guidelines, collectively explored the influence of aligner orthodontic treatment on masticatory muscles and associated systems. Notably, the identified studies focused on evaluating muscle activity, discomfort, and bite force in patients undergoing clear aligner therapy. This systematic review builds upon these foundational investigations by synthesizing existing evidence, offering a comprehensive analysis of the dynamic relationship between clear aligner therapy and the masticatory musculature. By addressing gaps in the current literature and highlighting the temporary nature of observed alterations, this review contributes valuable insights that underscore the need for further robust research in this evolving field. Therefore, this systematic review aims to synthesise the current scientific evidence on the relationship between aligner orthodontic treatment and the masticatory musculature and stomatognathic system.

## Materials and methods

### Eligibility criteria

The current review adhered to the PRISMA guidelines [43] for systematic reviews and meta-analysis, which formed the working framework for the study selection process. The review is applied for registration (509,370) in PROSPERO.

The PEOS protocol was framed to construct the research question, “Does intervention with aligners orthodontic treatment affect muscles of mastication and stomatognathic system?”

P: Human individuals, irrespective of age, gender, and ethnicity.

E: Exposure is the application of clear aligner orthodontic treatment intended to correct malocclusion and improve dental aesthetics.

C: No comparator group was included.

O: The outcome of interest was changes in masticatory muscle activity or temporomandibular disorders.

S: Clinical studies evaluating the relation or impact of aligners on outcome.

Clinical studies published only in English assessing the relation between aligner orthodontic treatment and masticatory anomalies, or TMDs, were included. Reviews, editorials, commentaries, case reports, and conference abstracts were excluded. Studies involving individuals with conditions that could independently impact the temporomandibular joint, such as rheumatoid arthritis or previous jaw trauma, were not considered. If the orthodontic treatment was not clearly defined or could not be classified as an aligner orthodontic treatment, the study was excluded.

### Search strategy

The search protocol was designed to identify all relevant studies in various databases. These databases included PubMed, Embase, Web of Science, Scopus, the Cochrane Library, CINAHL, PsycINFO, and Google Scholar. Index Terms used were ‘Clear Aligner Therapy’, ‘Masticatory Musculature’, ‘Stomatognathic System’ and ‘Study Design’. Search terms used for outcome were ‘Muscle Activity’, ‘Discomfort’, and ‘Bite Force’. The strategy was formulated using Medical Subject Headings (MeSH) terms and a boolean operator to ensure that all potential variations were included, as seen in Table 2.

**Table 2:** Database search protocol representation for this review

Database	Search String
PubMed	(“Aligner orthodontic treatment” OR “Clear aligner treatment” OR “Invisalign”) AND (“Masticatory anomalies” OR “Masticatory disorders” OR “Temporomandibular disorders” OR “TMD”) AND (“Systematic review” OR “Meta-analysis” OR “Review” OR “Clinical study” OR “Observational study” OR “Cohort study” OR “Case-control study” OR “Cross-sectional study” OR “Longitudinal study”)
Embase	(‘orthodontics’ OR ‘orthodontic appliances’ OR ‘dental braces’ OR ‘aligners’ OR ‘clear aligners’ OR ‘exp orthodontic appliances/‘ OR ‘exp orthodontic aligners/‘) AND (‘temporomandibular joint disorders’ OR ‘temporomandibular joint dysfunction syndrome’ OR ‘exp Temporomandibular Joint Disorders/‘ OR ‘exp Temporomandibular Joint Dysfunction Syndrome/‘)
Web of Science	TS=(“Aligner orthodontic treatment” OR “Clear aligner treatment” OR “Invisalign”) AND TS=(“Masticatory anomalies” OR “Masticatory disorders” OR “Temporomandibular disorders” OR “TMD”) AND TS=(“Systematic review” OR “Meta-analysis” OR “Review” OR “Clinical study” OR “Observational study” OR “Cohort study” OR “Case-control study” OR “Cross-sectional study” OR “Longitudinal study”)
Scopus	TITLE-ABS-KEY(“Aligner orthodontic treatment” OR “Clear aligner treatment” OR “Invisalign”) AND TITLE-ABS-KEY(“Masticatory anomalies” OR “Masticatory disorders” OR “Temporomandibular disorders” OR “TMD”) AND TITLE-ABS-KEY(“Systematic review” OR “Meta-analysis” OR “Review” OR “Clinical study” OR “Observational study” OR “Cohort study” OR “Case-control study” OR “Cross-sectional study” OR “Longitudinal study”)
Cochrane Library	(‘orthodontics’ OR ‘orthodontic appliances’ OR ‘dental braces’ OR ‘aligners’ OR ‘clear aligners’ OR ‘MeSH descriptor: [Orthodontic Appliances]’ OR ‘MeSH descriptor: [Orthodontic Aligners]’) AND (‘temporomandibular joint disorders’ OR ‘temporomandibular joint dysfunction syndrome’ OR ‘MeSH descriptor: [Temporomandibular Joint Disorders]’ OR ‘MeSH descriptor: [Temporomandibular Joint Dysfunction Syndrome]’)
CINAHL	((“Aligner orthodontic treatment” OR “Clear aligner treatment” OR “Invisalign”) AND (“Masticatory anomalies” OR “Masticatory disorders” OR “Temporomandibular disorders” OR “TMD”) AND (“Systematic review” OR “Meta-analysis” OR “Review” OR “Clinical study” OR “Observational study” OR “Cohort study” OR “Case-control study” OR “Cross-sectional study” OR “Longitudinal study”)
PsycINFO	(“Aligner orthodontic treatment” OR “Clear aligner treatment” OR “Invisalign”) AND (“Masticatory anomalies” OR “Masticatory disorders” OR “Temporomandibular disorders” OR “TMD”) AND (“Systematic review” OR “Meta-analysis” OR “Review” OR “Clinical study” OR “Observational study” OR “Cohort study” OR “Case-control study” OR “Cross-sectional study” OR “Longitudinal study”)
Google Scholar	“Aligner orthodontic treatment” OR “Clear aligner treatment” OR “Invisalign” AND “Masticatory anomalies” OR “Masticatory disorders” OR “Temporomandibular disorders” OR “TMD” AND “Systematic review” OR “Meta-analysis” OR “Review” OR “Clinical study” OR “Observational study” OR “Cohort study” OR “Case-control study” OR “Cross-sectional study” OR “Longitudinal study”

### Data extraction

Two reviewers did the data extraction separately. Any disagreements were addressed through discussion or consultation with a third reviewer. This rigorous process ensured the inclusion of only studies relevant to the research question, enhancing the reliability and validity of the review. Variables assessed were study ID, location, sample, age, gender ratio, and inferences.

### Risk of bias assessment

The methodological quality of the included studies was assessed using the Risk of Bias 2 (ROB-2) tool [44], which is widely recognised for evaluating the risk of bias in non-randomised studies. It provides a structured approach to assessing the quality and validity of each study included in the review. The tool assesses several key domains, such as bias arising from the randomization process, bias due to deviations from intended interventions, bias due to missing outcome data, bias in the measurement of outcomes, bias in the selection of the reported result, and overall bias.

## Results

### Literature search results

**Fig. 1:**
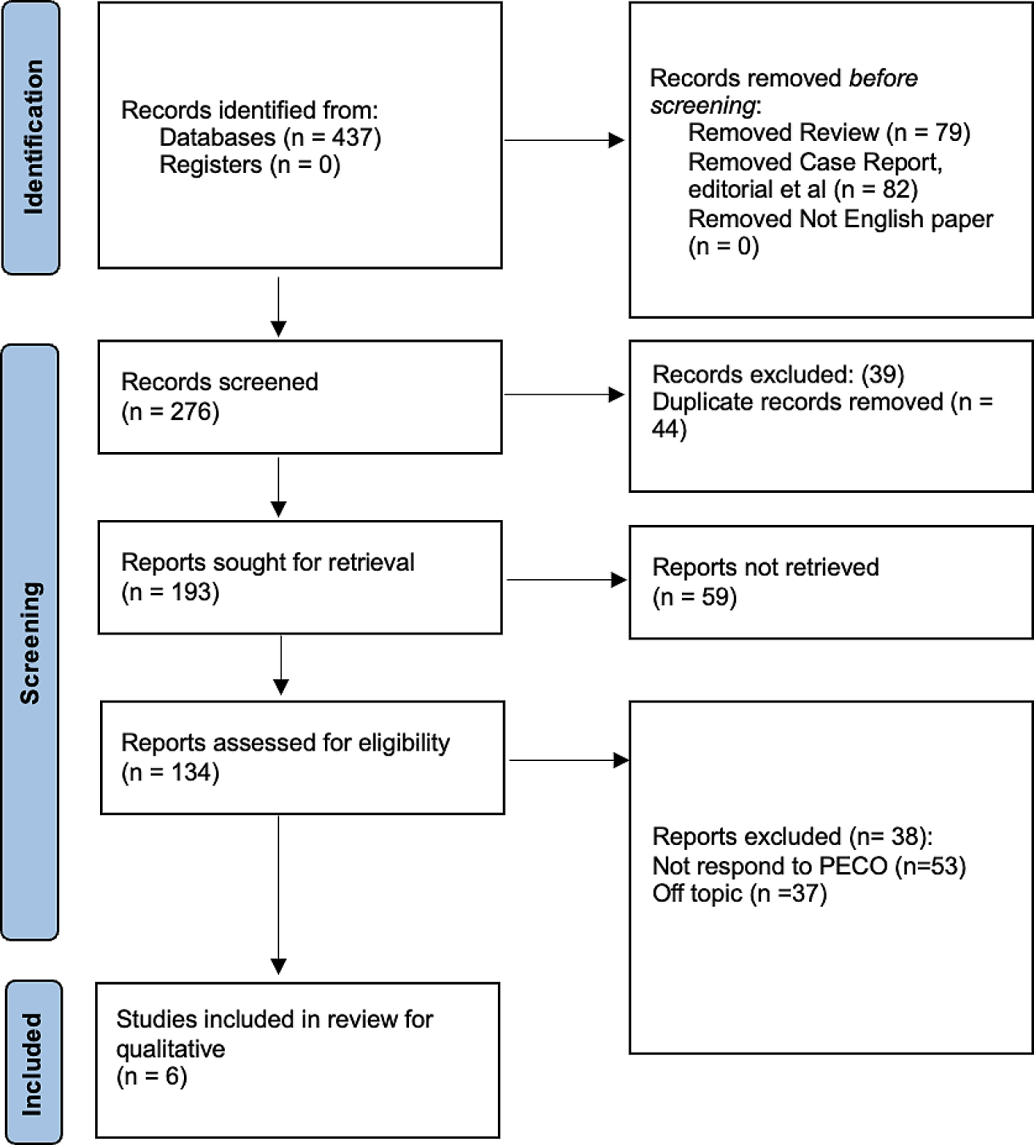
Prima Flowchart

An initial search was conducted in databases and registers, yielding a total of 437 records (Fig. 1). No records were identified through the registers. Before screening, 79 review articles and 82 case reports, editorials, and other non-research articles were removed from consideration. No records were excluded on the basis of language, as all the identified articles were in English. An additional 39 records were excluded for various reasons, and 44 duplicate records were removed. This left a total of 276 records for screening. Out of the 276 records that were screened, 193 were sought for retrieval. However, 59 of these could not be retrieved, leading to their exclusion. An additional 38 records were excluded for failing to respond to the PECO (Population, Exposure, Comparator, Outcome) criteria or for being off-topic, resulting in a further reduction of 51 and 37 reports, respectively. After these exclusions, 134 reports were assessed for eligibility. Following this assessment, only six studies [45–50] met the criteria and were included in the review for both qualitative and quantitative synthesis.

### Study characteristics

The demographic characteristics of the included papers [45–50] are represented in Table 3, whereas the in-depth assessments of those papers (in terms of assessed parameters and overall inferences) have been shown in Table 4. The included studies were conducted across various regions globally. The studies ranged from small sample sizes to larger cohorts, with the smallest sample size being 10 in a study conducted in Brazil [46] and the largest being 31 from a study in New Zealand [49]. The mean age of the participants varied across the studies, with the youngest mean age reported as 22.5 ± 3.5 years [45] and the oldest being 35.3 ± 17.6 years [49]. All studies predominantly involved females, with gender ratios favouring females over males. The follow-up timeframes for these studies also varied significantly. The shortest follow-up period was observed in a study from New Zealand, with a duration of only 9 days [49]. Conversely, a six-month follow-up period was reported in a study conducted in China [45]. Some studies did not specify the follow-up timeframe [46], which might have implications for the interpretation of the results, as the effects of orthodontic treatments can evolve over time, and a longer follow-up period might offer more comprehensive insights.

**Table 3:** Demographic variables pertaining to the included papers

Study ID	Region of study	Study year	Total sample size (number)	Mean age (years)	Gender ratio	Follow-up timeframe
Liu et al. [45]	China	2017	23	26.8 ± 2.4	All females	6 months
Manfrendini et al. [46]	Italy	2018	19	28.3 ± 2.4	14 females	Unspecified
Nota et al. [47]	Italy	2021	16	22.5 ± 3.5	8 females	3 months
Paes et al. [48]	Brazil	2023	10	29.9 ± 5.5	7 females	8 months
Pittar et al. [49]	New Zealand	2023	31	22 ± 4.3	17 females	9 days
Tran et al. [50]	Canada	2020	27	35.3 ± 17.6	22 females	1 month

**Table 4:** Technical characteristics pertaining to the included papers

Study ID	Study design	Parameters	Diagnostic criteria	Malocclusion category	Inference
Liu et al. [45]	A longitudinal study with three measurement points (T0, T1, T2).	- MMA - Parafunctional habits	- OBC - DC/TMD Axis II - sEMG	Not specified	- Temporalis muscle activity increased significantly between T0 and T1 (*P* < 0.05). - At MVC, the activities of the TA and SCM at T1 were significantly higher than those of T0 (*P* < 0.05) - OBC scores decreased greatly at the initial phase but then show a minor increase at further followup.
Manfrendini et al. [46]	Retrospective	- MMA during sleep	- sEMG.	Not specified	- Wearing the retainers did not significantly affect the sMMA variables.
Nota et al. [47]	Longitudinal study with three measurement points (T0, T1, T2).	- Mandibular elevator muscles activity - pain on palpation	- sEMG - RDC/TMD	Angle’s Class I malocclusion with crowding.	- The sEMG activity of masseter muscles at mandibular rest position showed a statistically significant reduction at T1 but returned to baseline levels at T2. - No changes were noted for sEMG activity at clenching position - At T0, pain was noted in 12.5% of the individuals which increased to 25% at T1.
Paes et al. [48]	Preliminary longitudinal study over an 8-month follow-up period.	- Biting force - Myoelectric activity of the superficial masseter and anterior temporal muscles.	- Surface Electromyography	Angle’s Class I and Class II malocclusion.	- sEMG increased to approximately 30% for RMS value at *p* = 0.001. - Bite force significantly reduced to < 20% at *p* < 0.05. - Clenching of the teeth were reported in higher percentages.
Pittar et al. [49]	Prospective	- MMA, - OD and - TMD symptoms in adults with different levels of self-reported oral parafunction.	-OBC for parafunctional habit - DC/TMD - Wireless EMG	- Individuals reported with parafunctional habits	- A decreased MMA was noted with a reduction in mean contraction episode amplitude at *p* = 0.003. - OD was increased at *p* = 0.048, particularly in high PFA subjects. - TMD symptoms were present throughout the evaluation phase in both groups
Tran et al. [50]	Multi-site prospective study with follow up of four weeks (	- Tooth pain - Masticatory muscle soreness - Pressure pain thresholds	- DC/TMD - VAS (100 mm guage)	- Class I and Class II malocclusion	- Muscle soreness was found in all phases, though the dummy phase showed significantly greater soreness than active phase. - Pain was present only in the first few days. Pain was significantly correlated with trait anxiety (*r* = 0.423; *p* = 0.008)

### Main findings

Clear aligners caused a short-term change from baseline (T0) to first month (T1) in muscle activity but reverted back to the T0 values on subsequent follow-ups [45, 47]. Synthesised evidence also suggested an increase in pain noted with clear aligner treatment [47, 50]. A significant reduction in bite force was also noted [48]. This suggests that an increase in muscle activity is only temporary because of occlusal changes. The study by Manfrendini et al. [46] did not show any significant changes in MMA activity throughout the study phase. Overall, it could be seen that wearing a clear aligner orthodontic appliance definitely impacts masticatory muscle activity and temporomandibular joints.

### Risk of bias

**Fig. 2:**
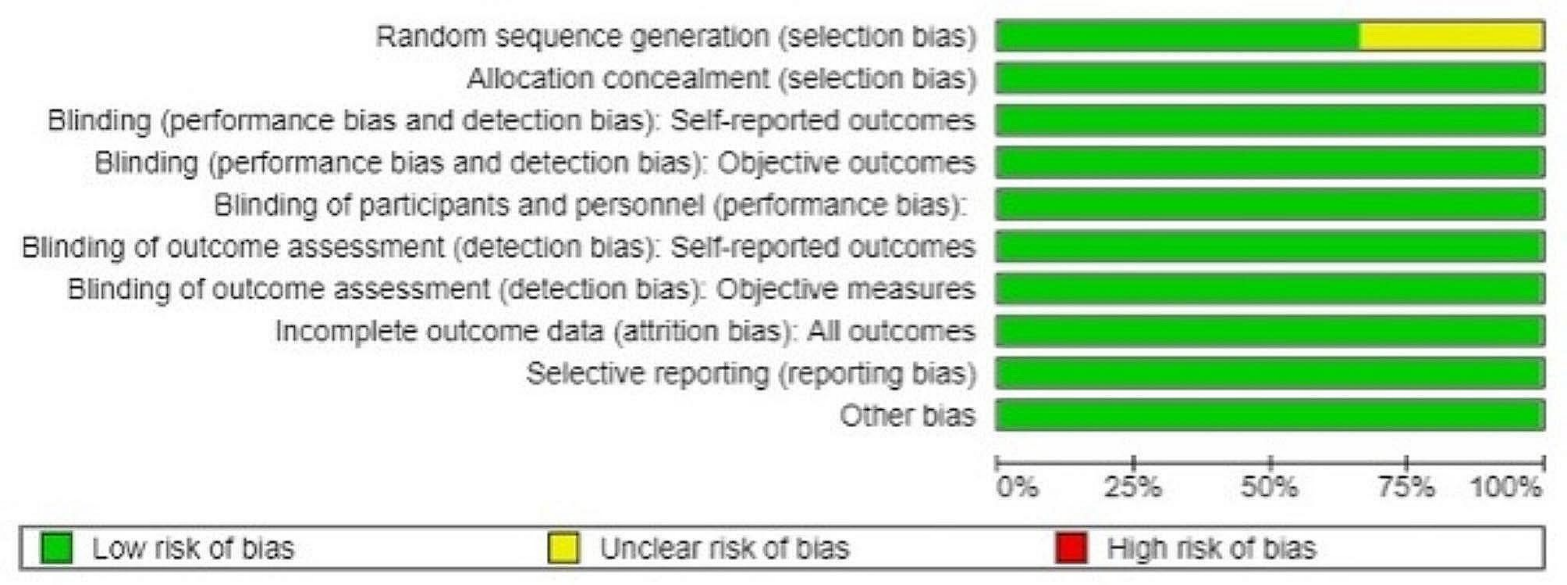
Risk of bias graph of studies included

**Fig. 3:**
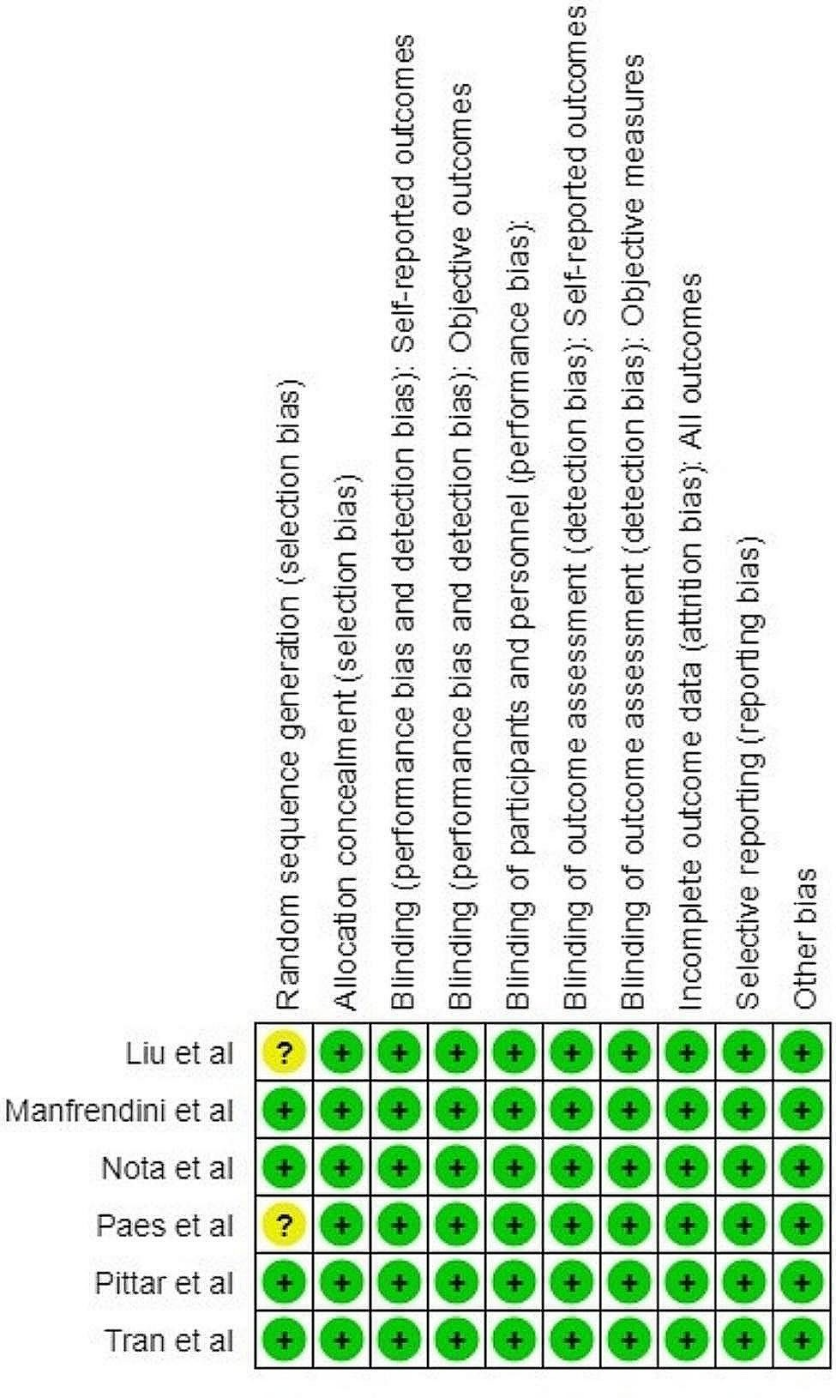
Risk of bias summary of studies included

Two authors performed the risk of bias assessment. Overall, the studies evaluated showed a low risk of bias. Liu et al. [45] were marked as having an “unclear” risk of bias in the selection domain considering all females were recruited for their study, and Paes et al. [48] were marked as having a small sample size (*n* = 10) (Figs. 2 and 3).

## Discussion

During the course of clear aligner therapy, there are temporary alterations in muscle activity and pain, primarily attributed to adaptation to the orthodontic appliance. Additionally, bite force tends to be reduced during this period [2, 3]. Clear aligner therapy requires patients to wear removable aligners that are designed to gradually shift teeth into their desired positions. As the aligners exert forces on the teeth, it’s common for individuals to experience temporary changes in muscle activity. This can manifest as muscle fatigue or tension as the masticatory muscles work to adapt to the new occlusal relationships and the forces exerted by the aligners. Temporary alterations are common during the adaptation process in clear aligner therapy. These changes, including muscle activity adjustments, often diminish as treatment progresses. However, they may lead to temporary pain and discomfort. Patients undergoing clear aligner therapy may experience mild and self-limiting soreness, especially in the masticatory muscles and the temporomandibular joint (TMJ). It’s crucial to counsel patients about the expected discomfort during this adjustment period.

During clear aligner therapy, it’s not uncommon for patients to report a reduction in their bite force. The primary reason for this decrease is the presence of aligners, which may affect the contact between the upper and lower teeth. As the teeth gradually shift, their occlusal surfaces may not engage as efficiently as they did prior to treatment. This can lead to a decrease in bite force, making it more challenging for patients to bite and chew effectively.

In the longitudinal study by Liu et al. [45], the impact of Invisalign treatment on the oral parafunctional behaviours and the electromyographic activities of masticatory muscles was evaluated. The research found that Invisalign treatment significantly decreased the OBC score and increased the activity of the anterior TA, indicating a relevant effect on the orofacial system. Manfrendini et al. [46] conducted a retrospective study to gauge the effects of invisible orthodontic retainers on the sMMA. The results indicated no significant difference in SB index or total number of masseter muscle contractions, suggesting that wearing or not wearing the retainers did not significantly affect the sMMA variables. Nota et al. [47] carried out a longitudinal study to assess mandibular elevator muscle activity and pain on palpation during the early stages of orthodontic treatment with clear aligners. The study found that while no statistically significant differences in muscular pain were observed, there was a significant reduction in sEMG activity of masseter muscles at mandibular rest position at T1, which returned to baseline levels at T2. Paes et al. [48] conducted a preliminary longitudinal study and found that the use of orthodontic aligners affected the biting force and myoelectric activity of the superficial masseter and anterior temporal muscles. Specifically, there was an increase in sEMG signal activity during the treatment, with a significant decrease in bite force.

The clear aligners of Invisalign treatment had a significant effect on the orofacial system in the study of Liu et al. [45]. The OBC score significantly decreased between T0 and T1, suggesting that aligners could reduce the incidence of parafunctional habits. The activity of the anterior TA increased significantly between T0 and T1 when measured at the MPP. During MVC, the activities of the TA and SCM at T1 were significantly higher than those at T0. But the muscle activity remained insignificant after 3 months, which the authors attributed to muscle plasticity. It was also the only study to recruit all females, quoting easier approachability. Nota et al. [47] employed only a single aligner system to eliminate any potential confounding factors. The increased muscle activity in the study of Paes et al. [48] was attributed to the aligner material (polyurethane) used for aligners, along with its physical properties and medium placement. Efforts to obtain appropriate jaw positioning also aggravate tooth clenching. It could contribute to the hypersensitivity of the masticatory muscles. A possible limitation of their study was that the authors did not mention the presence or absence of tooth contact in their study subjects. Pittar et al. [49] recruited samples who were students of a university attending orthodontic clinics and dichotomously categorised their patients into HPA and LPA. A merit of their study was that they assessed the levels of caffeine, alcohol, motorway space, and stress so as to eliminate confounding potential. But the examiners were not blinded by the group allocation. In the prospective study by Pittar et al. [49], it was determined that the wearing of PCAs was associated with a significant decrease in mean contraction episode amplitude in both groups. It was also observed that comfort levels increased and remained raised in all participants after the insertion of the PCAs, more so in the high-function group. Tran et al. [50] evaluated their patients in three phases: the passive phase and the active phase. The aligner fitting was perceived as more painful than the active phase. Though pain reduced, muscle soreness remained throughout the treatment (4 weeks) in their study. Age and occlusal characteristics were tested as confounders for muscle soreness, and no correlation was seen. The authors attributed muscle soreness to the clenching habit as a result of masticatory muscle adaptation to the appliance. Tran et al. [50] carried out a multi-site prospective study to investigate tooth pain and masticatory muscle soreness and tenderness in patients undergoing CAT. The study found that CAT caused mild tooth pain and masticatory muscle soreness, with both being affected by stress and trait anxiety.

The correlation between TMDs and orthodontic intervention has been the subject of numerous studies over the past decade [51, 52]. Despite the application of advanced and current diagnostic modalities such as magnetic resonance imaging and long-term follow-up scientific investigations [53], a consensus on this contentious topic remains elusive. Orthodontic intervention is not currently advocated as a therapeutic modality or as a strategy to mitigate the risk of these disorders, owing to a lack of substantial evidence linking orthodontic treatment to an elevated predisposition to TMDs and occlusal alterations [54, 55]. Nonetheless, the clinical management strategies employed prior to and during orthodontic treatment have undergone transformation due to an enhanced focus on TMD signs and symptoms [56]. Moreover, while TMDs typically present a cyclical pattern of manifestations and often exhibit spontaneous improvement without intervention, the treatment of these conditions necessitates a multidisciplinary approach and robust protocols [57, 58].

The prevailing sentiment among the majority of orthodontists is that orthodontic intervention does not significantly influence TMD symptoms. This aligns with the outcomes of most prior studies, which suggest that orthodontic treatment neither prevents nor precipitates TMDs [59–61]. However, in contradiction to the findings reported by a literature review [62], which proposed that orthodontic intervention does not augment the risk of developing TMD signs and symptoms, irrespective of the therapeutic technique employed and the extraction status, professionals in oral surgery and oral medicine expressed divergent views.

In retrospect, our review mirrored several findings from the reviews conducted by both Leite et al. [62] and Fernandez et al. [52], while also highlighting a few discrepancies. In alignment with the findings of Leite et al. [62], our review also explored the relationship between TMD and orthodontic treatment and/or malocclusion through the lens of the most recent studies conducted over the last 15 years. The shared objective was to ascertain whether orthodontic intervention augments the incidence of TMD signs and symptoms and if it can be recommended as a therapeutic or preventive measure for TMD. Both our review and that of Leite et al. [62] found that the studies linking TMD signs and symptoms to orthodontic treatment yielded inconsistent results. While some studies reported positive effects of orthodontic treatment on TMD signs and symptoms, none were able to demonstrate a statistically significant difference. In concordance with Leite et al.‘s findings [62], our review also concluded that orthodontic treatment did not pose a risk to the development of TMD signs and symptoms, irrespective of the treatment technique, premolar extraction status, and the patient’s pre-existing malocclusion type.

Similarities were also observed when comparing our review to that of Fernandez et al. Fernandez et al.‘s review [52] aimed to evaluate the potential association between malocclusions, orthodontic treatment, and the development of TMD. In agreement with their review, our evaluation also found that the articles establishing a link between orthodontic treatment and the development of TMD produced drastically varied results. Some suggested that orthodontic treatment could ameliorate TMD signs and symptoms, yet none yielded statistically significant differences. Corroborating the findings of Fernandez et al. [52], our review also found no evidence to support a cause-and-effect relationship between orthodontic treatment and TMD or that such treatment could prevent or improve the condition.

In contrast, where the reviews diverged was in the evaluation of the role of orthodontic treatment in the management of TMD. While both Leite et al. [62] and Fernandez et al. [52] did not find a statistically significant effect of orthodontic treatment on TMD symptoms, our review suggested that the efficacy of orthodontic treatment in managing TMD might be contingent on the specific orthodontic treatment modality employed. For example, evidence from our review suggested that the use of mandibular advancement appliances might effectively mitigate TMD symptoms. However, this point was not explored or mentioned in the studies conducted by Leite et al. [62]. and Fernandez et al. [52].

Further, our review’s findings and those of Langaliya et al. [63], Ivorra et al. [64], and Alam et al. [65] also reveal a blend of parallel and divergent interpretations. Langaliya et al.‘s review [63] investigated the long-term influence of mandibular advancement devices (MAD) used in obstructive sleep apnea (OSA) treatment on TMD. In line with some of our observations, they found that certain studies reported a significant reduction in TMD symptom severity and frequency post-MAD treatment. However, other studies did not observe significant changes in TMD symptoms or TMJ-related parameters from baseline to follow-up intervals, mirroring our findings of inconsistent results in this area. Our review results are similar in that a temporary increase in TMJ-related pain or symptoms at the start of the follow-up period was reported, which later subsided. Crucially, no OSA patient discontinued MAD due to TMDs in their review, a finding that was not directly explored in our review.

Ivorra et al.‘s [64] review examined the main effects on the TMJ of using functional appliances in healthy patients and those with pre-existing disorders. Their findings that the condyle was found to be in a more advanced position post-treatment, with condyle remodeling and adaptation of the glenoid fossa’s morphology, were not directly addressed in our review. However, their observation that no significant adverse effects on the TMJ were seen in healthy patients and that the appliances could improve joints that initially presented forward dislocation of the disc echoed our findings that orthodontic treatment did not pose a risk to the development of TMD signs and symptoms. Alam et al.‘s review [65] aimed to evaluate the influence of TMDs on orthodontic management and explore the association between TMDs and various aspects of orthodontic treatment. Their meta-analysis indicated a significant overall effect, suggesting orthodontic treatment might increase the risk of developing TMD, a finding not supported by our review. Additionally, they found that TMD patients had higher odds of experiencing orthodontic issues than those without TMD and that orthodontic treatment could negatively impact the psychological well-being of TMD patients. This aspect was not explored in our review, highlighting a potential area for future investigation.

Despite its insightful findings, this systematic review had several limitations that warrant consideration. The number of studies that met the inclusion criteria was relatively small, with only eight studies included in the final analysis. This limitation might have reduced the statistical power to detect significant associations and also restricted the scope of the review’s conclusions. Also, there was a significant heterogeneity among the included studies in terms of their design, sample size, duration of follow-up, and outcome measures. This heterogeneity made it difficult to compare and consolidate the results across studies, potentially obscuring more nuanced relationships between the assessed correlations. Moreover, the review was based on published studies, which could introduce publication bias, as studies with non-significant or negative results are less likely to be published. This review did not include grey literature, which might have provided additional insights. The availability of limited data across studies hindered the creation of a homogeneous dataset necessary for a meaningful meta-analysis. Variations in reporting and data collection methods limited the ability to perform a pooled analysis.

## Conclusion

The impact of clear aligner therapy on the masticatory musculature and the stomatognathic system is a crucial consideration in orthodontic care. The main findings indicated that clear aligners caused a short-term change in muscle activity, which reverted to baseline values in subsequent follow-ups. Pain notably increased with clear aligner treatment, and a significant reduction in bite force was observed. This transient increase in muscle activity aligns with the occlusal changes introduced by the aligners. Notably, one study did not show significant changes in muscle activity throughout the study phase. The risk of bias assessment indicated an overall low risk across the included studies. However, uncertainties in the selection domain and small sample sizes were identified in specific studies. The review highlighted the need for clinicians to consider the potential impact of aligner treatment on TMDs during the treatment planning process and to adequately inform patients about possible outcomes. Despite the insights gained, the review also revealed gaps in the existing literature, indicating a need for more robust, high-quality research to fully understand the implications of aligner treatment on TMDs.

## Data Availability

The data will be available on reasonable request from the corresponding author.
